# A Case of Prolonged Fever in a Patient Infected With COVID-19 on Ofatumumab

**DOI:** 10.7759/cureus.43274

**Published:** 2023-08-10

**Authors:** Yasin Uddin, Hector Ramirez, Monish A Sheth

**Affiliations:** 1 Internal Medicine, Baylor Scott & White Medical Center, Temple, USA; 2 Infectious Diseases, Baylor Scott & White Medical Center, Temple, USA

**Keywords:** coronavirus disease 2019 (covid-19), sars-cov-2, immunocompromised patient, paxlovid, relapsing-remitting multiple sclerosis (rrms), sars-cov-2 infection, anti-cd20 monoclonal antibodies, high fever, ofatumumab, kesimpta

## Abstract

We discuss a case of a 53-year-old woman with multiple sclerosis on monthly ofatumumab injections, who was infected with SARS-CoV-2 with persistent fevers for seven weeks. She was hospitalized for fever with diagnostic workup being unremarkable with negative SARS-CoV-2 IgM and undetectable nucleocapsid IgG antibodies four weeks out from the initial infection, indicating she may not have mounted an appropriate immune response to the infection. Patients on immunosuppression therapy may have a prolonged course of disease given that medications such as ofatumumab can take up to 24 weeks of B-cell recovery post-treatment discontinuation and a longer road to recovery.

## Introduction

Prolonged symptoms reported by patients infected with COVID-19 include fatigue, dyspnea, parosmia, cognitive impairments, headaches, memory impairments, insomnia, chest discomfort, and even anxiety [1]. Persistent and prolonged fevers greater than 101°F were not typically reported. Here, we report a case of a 53-year-old woman receiving ofatumumab (Kesimpta) for multiple sclerosis (MS) with recent use of Paxlovid for initial treatment of COVID-19 who presented with prolonged fevers. The objective of this case is to describe the presenting symptoms and diagnostic workup of an MS patient with fever infected with SARS-CoV-2 and to investigate whether her prolonged symptoms are due to her immunocompromised status worsened by anti-CD20 monoclonal antibody treatment in the setting of COVID-19, rebound infection due to recent use of Paxlovid, or an alternate cause.

## Case presentation

A 53-year-old woman with a past medical history of MS, Crohn’s disease, hypertension, vitamin B12 deficiency, and anemia presented to the hospital with three weeks of fever (as high as 104°F), chills, myalgias, generalized weakness, and shortness of breath that has progressively been worsening. She was evaluated by her primary care physician (PCP) and diagnosed with COVID-19 one week prior to hospitalization with a SARS-CoV-2 antigen test and treated with Paxlovid, prednisone, azithromycin, and augmentin. Despite treatment, she remained symptomatic.

**Figure 1:**
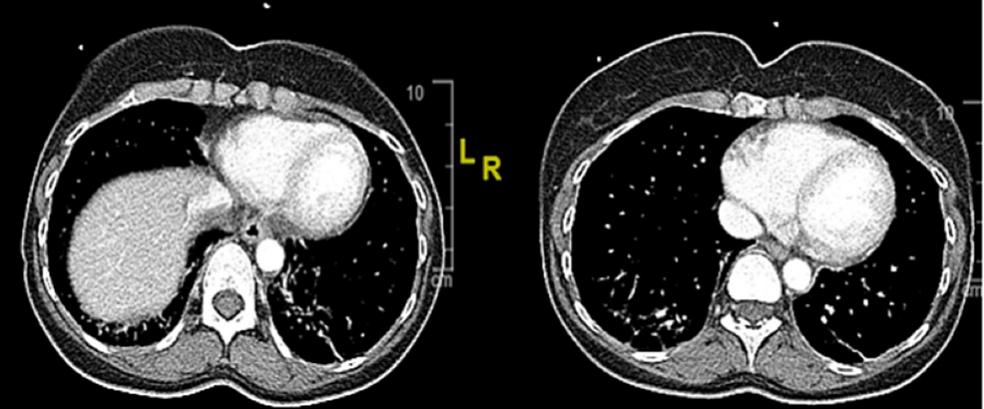
Axial chest computed tomography showing patchy opacities posterior within both lower lobes.

She was on a monthly injection of ofatumumab for the treatment of her relapsing-remitting MS for years after failing numerous disease-modifying drugs (DMDs) and was advised by her neurologist to delay restarting two weeks after her symptoms improved. Her Crohn's disease has been in remission for the last 30 years and is not on any medication for that. The physical examination revealed an ill-appearing woman with a body mass index of 21.59 kg/m2. Her vital signs were notable for blood pressure of 141/81 mm Hg, tachycardic to 110 beats/minute, respiratory rate of 20/minute, temperature of 103°F, and saturating at 99% on room air. She still tested positive for COVID-19 with SARS-CoV-2 antigen testing on day 13 of her illness and again on days 20 and 22 with repeated testing with SARS-CoV-2 polymerase chain reaction via nasopharyngeal swab. Her labs showed an elevated C-reactive protein (CRP) level of 23.6 mg/L (reference: 0.0-3.2 mg/L) on admission. Pertinent negative workup included flu/respiratory syncytial virus (RSV), antinuclear antibody (ANA)/antineutrophil cytoplasmic antibody (ANCA), Rocky Mountain spotted fever (RMSF) serologies, *Ehrlichia* panel, beta-D-glucan, *Cryptococcus* antigen (Ag), *Histoplasma* serum Ag, *Coccidioides* serum Ag, malaria smear (x2), and blood culture (x2). SARS-CoV-2 IgM and nucleocapsid IgG antibodies were negative. Chest computed tomography (CT) revealed patchy opacities posteriorly within both lower lobes reflecting sequela of known resolving COVID-19 pneumonia (Figure 1).

The patient received vancomycin and piperacillin-tazobactam until blood cultures returned negative 48 hours later. Due to persistent high-grade fevers and concerns for tick-borne diseases, she was started on doxycycline for five days until her workup returned negative. She reported mild subjective improvement in her symptoms except for fever. After an extensive workup, her fevers were attributed to the COVID-19 infection and was discharged home with advice to take Tylenol (acetaminophen) for symptomatic relief, as she was still reporting fevers at discharge. At the PCP visit one week after discharge, she reported fevers of up to 101°F, and at the neurology follow-up three weeks after discharge, she reported being afebrile for a week, which culminated into seven weeks of persistent fevers.

## Discussion

Current evidence describes patients with comorbidities, including but not limited to diabetes, obesity, asthma or chronic lung disease, and sickle cell disease, or those who are immunocompromised are at an increased risk of becoming severely ill from COVID-19 with long-lasting complications [2]. Of that vulnerable population are patients with relapsing-remitting MS on biologic therapy.

The initial workup in our patient was negative, and SARS-CoV-2 IgM and nucleocapsid IgG antibodies were also notably negative. This was profound mainly because IgM levels were found to be increased during the first week after SARS-CoV-2 infection, peaked for two weeks, and then reduced to near-background levels in most patients. IgG was detectable after one week and peaking at roughly day 25 and was still maintained at a high level even after four weeks of infection [3]. Given that her IgM and IgG antibodies were negative almost four weeks out from the initial infection indicates that she may not have mounted an appropriate immune response to the infection [4]. Differentials subsequently included rebound COVID-19 infection due to the patient’s previous use of Paxlovid or persistent infection in the setting of ofatumumab. Some patients who were treated with Paxlovid experienced rebound COVID-19 infections and symptoms two to eight days after completing a five-day course of Paxlovid [5], but our patient had continued symptoms.

Ofatumumab is believed to work by selectively binding to sites on both the small and large extracellular loops of CD20. When delivered subcutaneously, it promotes preferential depletion of B cells in the lymph nodes shown to be associated with disease activity in MS [6,7]. Additional DMDs such as rituximab and ocrelizumab with adverse outcomes related to COVID-19, including hospitalization and intensive care unit admission, were studied and revealed higher risks of hospitalization and ICU admission among these DMDs. Ofatumumab may also take up to 24 weeks for B cell recovery post-treatment discontinuation, so this may have been a contributing factor in the prolonged nature of her symptoms, given that her last date of treatment was four weeks prior to infection and symptom onset [8].

This patient’s prolonged symptoms were due to ofatumumab and not a relapse or rebound. Careful attention should be directed to patients on DMDs regardless of vaccination status because of impaired immunity leading to prolonged diseases and the increased risk of associated mortality [9].

## Conclusions

Numerous research studies have shown that various patients with several comorbidities infected with SARS-CoV-2 have variable long-term presentations. Our patient with relapsing-remitting MS on monthly injections of the anti-CD20 therapy ofatumumab had persistent symptoms of prolonged fevers as high as 103°F. Though she completed a five-day course of Paxlovid for treatment, her symptoms persisted. It was determined that her symptoms were not rebound infection as her symptoms started even prior to her brief Paxlovid use. The workup in the hospital was unremarkable except for SARS-CoV-2 IgM and nucleocapsid IgG antibodies, which were negative almost four weeks out from the initial infection. This piece of data was monumental as this indicated that she may not have mounted an appropriate immune response to the initial infection, as she was on anti-CD20 therapy, which suppressed her B-cell activity for adequate immune function and propagated her symptoms due to impaired natural innate and humoral immunity response.
